# Advances in Bio-Optical Imaging for the Diagnosis of Early Oral Cancer

**DOI:** 10.3390/pharmaceutics3030354

**Published:** 2011-07-11

**Authors:** Malini Olivo, Ramaswamy Bhuvaneswari, Ivan Keogh

**Affiliations:** 1 School of Physics, National University of Ireland Galway, Ireland; 2 Department of Pharmacy, National University of Singapore, No. 18 Science Drive 4, Block S4, 117543, Singapore; 3 National Cancer Centre Singapore, 11 Hospital Drive, 169610, Singapore; E-Mail: dmsram@nccs.com.sg; 4 Consultant Otolaryngologist, Head & Neck Surgeon, Academic Department of Otorhinolaryngology, National University of Ireland Galway & Galway University Hospitals, Ireland; E-Mail: ivan.keogh@nuigalway.ie

**Keywords:** oral cancer, optical imaging, fluorescence diagnosis, confocal endomicroscopy, surface enhanced Raman spectroscopy, optical coherence tomography, confocal reflectance microscopy

## Abstract

Oral cancer is among the most common malignancies worldwide, therefore early detection and treatment is imperative. The 5-year survival rate has remained at a dismal 50% for the past several decades. The main reason for the poor survival rate is the fact that most of the oral cancers, despite the general accessibility of the oral cavity, are not diagnosed until the advanced stage. Early detection of the oral tumors and its precursor lesions may be the most effective means to improve clinical outcome and cure most patients. One of the emerging technologies is the use of non-invasive *in vivo* tissue imaging to capture the molecular changes at high-resolution to improve the detection capability of early stage disease. This review will discuss the use of optical probes and highlight the role of optical imaging such as autofluorescence, fluorescence diagnosis (FD), laser confocal endomicroscopy (LCE), surface enhanced Raman spectroscopy (SERS), optical coherence tomography (OCT) and confocal reflectance microscopy (CRM) in early oral cancer detection. FD is a promising method to differentiate cancerous lesions from benign, thus helping in the determination of adequate resolution of surgical resection margin. LCE offers *in vivo* cellular imaging of tissue structures from surface to subsurface layers and has demonstrated the potential to be used as a minimally invasive optical biopsy technique for early diagnosis of oral cancer lesions. SERS was able to differentiate between normal and oral cancer patients based on the spectra acquired from saliva of patients. OCT has been used to visualize the detailed histological features of the oral lesions with an imaging depth down to 2–3 mm. CRM is an optical tool to noninvasively image tissue with near histological resolution. These comprehensive diagnostic modalities can also be used to define surgical margin and to provide a direct assessment of the therapeutic effectiveness.

## 1. Introduction

At 2008, worldwide incidence of oral cancer was numbered at 263,020 cases, and the number of deaths totaled to 127,654. Though oral cancer accounted for only just above 2% of overall cancer incidences, its mortality rate of approximately 50% is considered one of the serious concerns among all pathologies [1]. Oral cancer is the 6th most common cancer in the world [2]. Similar to other types of cancer, low 5-year survival rate of less than 50% can be attributed to detecting the carcinoma only at its advanced stages. This fact can be emphasized for oral cancer, as the prognosis of patients with oral cavity cancer has remained poor, even with the improvements in diagnostic and therapeutic modalities, along with easy accessibility of oral cavity. Thus, early detection and diagnosis of neoplastic changes in the oral cavity would pave the way for better prognosis.

Currently, first line of screening of oral cavity abnormalities is performed by visual inspection, which is subjective. Clinical endoscopic examination and invasive needle biopsies followed by histopathological analysis remains the gold standard for diagnosis and surveillance of oral cavity cancer. However, these conventional techniques has their own limitations such as: (i) difficulty to distinguish benign from malignant lesions; (ii) difficulty in determining margin of lesions; (iii) can be subjective, especially during histopathological analyses; and also (iii) tissue biopsies can be invasive and painful for the patients. Computer analysis (Oral CDx) of brush biopsies have also been tried in clinical studies, but this again proved inadequate for mass screening, as brush biopsies are again subjective, and suffers from all aforesaid limitations. Considering these facts, it is imperative that a new rapid and accurate diagnostic method for early oral cavity cancer detection is much needed to reduce the resulting mortality rate. Given the difficulty of detecting oral cancer early [3] and the prevalence in developing nations [1], any technique that improves the diagnosis should also improve the screening ability among large population. Therefore, optical techniques that are robust, accurate, of low cost, portable and easy to handle can be used effectively for clinical applications.

During carcinogenesis in the oral cavity, structural and biochemical changes occur in both the epithelium and stroma, altering the optical and biological properties of dysplastic and cancerous tissue. These changes acts as molecular signatures which can be detected in early oral cancers, using optical principles like endogenous (autofluorescence) and exogenous fluorescence. Autofluorescence analysis makes use of the intrinsic fluorescing ability of biomolecules, like NADH, FAD, *etc.* in epithelial layers, and elasin and collagen in stroma, when excited by UV/Visible radiation of suitable wavelength. Carcinogenesis would induce quantitative and/or qualitative changes to native fluorophores, which can be detected by capturing its emission signal. Apart from native fluorophores, fluorescence signals can also be obtained by adding external fluorophores, like 5-aminolevulinic acid (5-ALA), hypericin, *etc.* Molecular imaging probes such as gold nanoparticles, iron oxide nanorings and other biomarker targeted conjugates play an important role in optical imaging. In addition to this, novel endoscopic optical imaging modalities such as laser confocal endomicroscopy [4] and optical coherence tomography (OCT), can be employed to acquire *in vivo* high resolution imaging of oral epithelial tissues for diagnostic purposes. Presence of cancer biomarkers can also be detected in early oral cancers by using surface enhanced Raman spectroscopic (SERS) imaging that analyzes distinct scattering signal from specific antibody conjugated gold nanoparticles labeled with highly SERS efficient reporter tags [5]. Confocal reflectance microscopy (CRM) can image viable tissue with high-resolution and contrast without performing a biopsy and processing the tissue, as in standard histology. This review provides description of each of these modalities, mainly in the context of early detection of oral cavity cancer.

## 2. Probes for Molecular Imaging

Molecular imaging is a promising non-invasive modality that can image and quantify molecular changes associated with diseases. It can be used for the early detection of malignancies, staging tumors, and also for monitoring the efficacy of treatment. Three important elements are needed for molecular imaging that includes a labeled probe that can be detected with high sensitivity and a ligand that has high affinity and specificity to the target, a method to amplify the signal from the label and a high-resolution imaging modality to detect the label.

Gold nanoparticles have been extensively utilized in cellular imaging because of their favorable physical and chemical properties [6]. Kah *et al.* [5] have demonstrated the potential of antibody conjugated gold nanoparticles to target and illuminate cancer cells under a reflectance-based optical imaging system. It has been shown that gold nanoparticles can provide an optical contrast to discriminate between cancerous and normal cells and their conjugation with antibodies allows them to map the expression of relevant biomarkers for molecular imaging. Another exciting new aspect is to exploit gold nanoparticles as multifunctional SERS nanosensors. These mobile sensors can probe cellular chemistry at subendosomal resolution. Because of the large effective Raman scattering cross section, SERS probes fulfill the requirements of dynamic *in vivo* systems that is the use of very low laser powers and very short data acquisition times [7]. Among the different types of gold nanostructure, the efficiency of gold nanoshells as an exogenous contrast agent for optical imaging of cells is well documented [8]. Gold nanoshells are a type of composite spherical nanoparticles consisting of a dielectric core, e.g., silica coated with a thin gold layer. These nanoparticles exhibit strong light scattering, with scattering cross sections several times the particle geometric cross section in the near infrared (NIR) due to the strong plasmon resonance of the metallic–dielectric concentric spherical configuration [9]. By varying the ratio of core size to shell thickness, the nanoparticles' peak plasmon resonance can be systematically tuned across a broad range of the optical spectrum from the visible to NIR [10], which includes the 750–800 nm optical window for biomedical imaging. On the other hand, iron oxide nanoparticles are particularly useful as MRI contrast agents because of their strong magnetic properties. MRI iron-oxide nanoparticles are being clinically used to image the liver or diagnose metastatic lymph nodes [11] and are becoming the basis for many MRI molecular imaging probes.

Molecular imaging modalities offer potential for assessing angiogenic responses and monitoring therapeutic angiogenesis. Vascular endothelial growth factor (VEGF) signaling pathway plays a pivotal role during the development of the normal vasculature and many disease processes and is a main target for emerging anti-angiogenic therapies [12,13]. Therefore VEGF is an attractive target for molecular imaging because it is overexpressed in angiogenic endothelium [14] thus allowing contrast agents to accumulate and be retained. A single-chain recombinant VEGF expressed with a cysteine-containing tag can allow site-specific labeling with contrast agents for near-infrared fluorescence imaging, single-photon emission computed tomography or positron emission tomography [15]. These probes retain VEGF activities *in vitro* and undergo selective and highly specific focal uptake into the vasculature of tumors and surrounding host tissue. *In vivo* molecular imaging with specific targeting of VEGF using single chain VEGF based probe is also possible in murine tumors, human xenografts and tissue specimens using laser confocal endomicroscopy. In another study, 99mTc or Cy5.5-labeled chitosan-DC101 conjugates have shown the potential to be useful as VEGFR-2-targeted imaging agents for monitoring ischemia [16]. In addition to that, radiolabeled VEGF has been developed as a single-photon emission computed tomography tracer for imaging solid tumors and angiogenesis in humans [17,18].

Due to an increased expression of VEGF in most aggressive tumors, it is important to measure VEGF levels in the tumors to design better anti-cancer treatment protocols. Bevacizumab is a humanized antibody against VEGF and it binds to all VEGF isoforms. Bevacizumab is approved for clinical use in metastatic colon carcinoma and non-small cell lung cancer. Alexa Fluor 680, that is a NIR fluorescent dye with absorbance maximum at 684 nm and emission maximum at 707 nm has shown potential in VEGF imaging when conjugated with Bevacizumab [19]. On the other hand, integrin alphabeta3 provides numerous possibilities for diagnostic imaging probes and therapeutic potentials for angiogenesis research. Additionally, ligand array of integrin antagonists on nanoparticles has proven to be a viable strategy to target vascular surface receptors on endothelial cells [20]. These novel targeted approaches for imaging angiogenesis can play a crucial role for evaluation of therapeutic interventions to promote angiogenesis. Therefore, recent advances in molecular probes have made it an excellent tool to diagnose and treat diseases in a non-invasive way. Next, we will focus on certain novel optical imaging techniques that have been useful in the diagnosis of early oral cancers.

## 3. Autofluorescence

When oral cavity tissue is illuminated with light in the UV-Visible region, fluorophores absorbs a portion of photons and gets excited, which then emit lower energy photons that can be detected as fluorescence from the mucosal surface. Carcinogenesis changes the concentration and fluorescence emitting properties of these native fluorophores; detecting and quantifying this change in oral cavity can enable us to diagnose and find the severity of the cancer. Autofluorescence analyses of tissues can be performed by obtaining the autofluorescence spectral analysis or autofluorescence imaging. Spectral analysis also called autofluorescence spectroscopy can be used for the classification of lesions. In addition to that, the autofluorescence emitted in the tissue can be imaged using a CCD camera - called autofluorescence imaging. Though autofluorescence spectroscopy could classify normal from pre-malignant and malignant lesions [21] with good accuracy, its inability to demarcate the area of lesions, because of the small sampling size, makes it laborious to scan the entire oral cavity; therefore only autofluorescence imaging, with sampling area of several square centimeters [21] and also fits into the scope of this review, will be considered further.

Early work on analyzing autofluorescence in oral mucosa, by Onizawa *et al.*, detected fluorescence from malignant lesions, between orange and red part of the spectrum; this study suggested that the concentration of blood constituents along with protoporphyrin, are responsible for this autofluorescence, which seemed to increase during tumor progression and decrease during tumor regression [22]. Subepithelial stromal collagen fibers are the predominant source of autofluorescence in oral mucosa [23]. These are confirmed in some fluorescence studies which reported decreased green fluorescence, attributed to collagen degradation, and increased red fluorescence, attributed to release of porphyrin [24,25]. This change in fluorescence signal between normal and neoplastic lesion was confirmed in oral cavity lesions and the possibility of delineating tumor margin based on this fluorescence change was studied by Poh *et al.* [26]. They illuminated oral cavity with blue light from a hand-held device, which was later named as VELscope, and visualised autofluorescence. Loss of green fluorescence in cancerous tissues was noted and physicians manually demarcated the tumor margins based on this green autofluorescence change, and the microdissected tumor margin biopsies underwent Loss of Heterozygosity (LOH) analysis to confirm the demarcation. This hand-held device underwent three successful clinical studies and was approved by Food and Drug Administration (FDA). This shows that simple non-invasive autofluorescence detection can effectively detect even occult neoplastic lesions, which might otherwise go unnoticed using white light visualization.

Another important study by Richards-Kortum *et al.* quantified the green-red autofluorescence using digital image processing and delineated areas of oral dysplasia and carcinoma [27]. This study reported that autofluorescence obtained by illumination of tissues with light of 405 nm wavelength produced highest discrimination. They calculated the ratio of red-to-green intensity for each pixel digitally within the autofluorescence image, and classified neoplastic with non-neoplastic tissues based on a set threshold value. Disease probability maps, which represent areas where probability of tissues being neoplastic are high, were generated based on this ratio. These regions were confirmed with histological analysis, and validation of this technique resulted in 100% sensitivity and 91.4% specificity.

The experimental setups reported for autofluorescence imaging of oral cavity are simple and similar. Autofluorescence images are obtained by exciting tissues generally with light in 375–440 nm range [21], but the aforementioned study by Richards-Kortum, *et al.* reported that excitation at 405 nm best discriminates normal with cancerous lesions [27]. A wide-field optical microscope is used to collect digital autofluorescence images with a colour CCD camera. Patients are usually imaged in outpatient settings or in the operating room under mild anesthesia.

The parameter most of the autofluorescence imaging studies used is the weakened green fluorescence, suspected from connective tissue degradation, and increased red fluorescence, suspected from accumulated porphyrin. Though few studies reported that red fluorescence is not really specific for malignancies [28], the ratio of red to green autofluorescence was still found to be a good classifier. Though lot of studies reported autofluorescence imaging to be an objective technique for the detection and demarcation of neoplastic oral cavity lesions [21,27,29], finding its capability of detecting invisible tumors within high-risk populations, such as heavy tobacco users, patients with history of oral cavity cancer, *etc.*, should be the next step for consideration as robust screening technique. VELscope has provided the precedent for clinically viable autofluorescence viewing of oral lesions, but a robust system to quantify the autofluorescence and be able to stage tumors should be the way forward. Though studies by Richards-Kortum, *et al.* did quantify autofluorescence through ratio of green-to-red fluorescence, more extensive studies with wide disease prevalence must be undertaken, in order to classify benign and malignant lesions, and to be considered for screening large population.

Evaluation of autofluorescence imaging with VELscope can assist in the identification of malignant and potentially malignant oral lesions from normal mucosa in high-risk patients but does not help in discriminating benign lesions from malignant or premalignant mucosal conditions [30]. Another study has also shown that VELscope interpretation did not enhance or alter the clinical management of the suspicious lesions. The study also reported that several commonly occurring conditions, such as mucosal pigmentations, ulcerations, irritations, and gingivitis were associated with a loss of fluorescence using VELscope [31]. On the other hand, Fluorescence lifetime imaging (FLIM) when applied to intrinsic tissue autofluorescence can directly contrast a range of surface tissue tumors, including gastrointestinal (GI) tissues, using compact, clinically deployable instrumentation achieving wide-field fluorescence lifetime images of unprecedented clarity [32]. However, one of the major disadvantages of autofluorescence imaging is still the low specificity in detection of premalignant lesions and early-stage cancer. This disadvantage could be overcome with the appearance of new and improved technologies in autofluorescence, such as the addition of backscattered light analysis, ultraviolet spectra, fluorescence-reflectance or dual digital systems [33].

Only when screened early for oral cancer risks, intervention is possible and the treatment would be more effective. In this regard, autofluorescence imaging comes close to being used as a robust oral cavity cancer screening and detection technique. Early detection of small cancer foci is required to improve the survival rate. Here, autofluorescence (AF) might become useful as *in vivo* biochemical changes of cancer metabolism can be shown by fluorescence. However, several non-dysplastic lesions may be non-fluorescent and occasional false positive results have been reported. Therefore fluorescence agents are being explored to further improve oral cancer diagnostics.

## 4. Fluorescence Diagnosis

**Figure 1.**
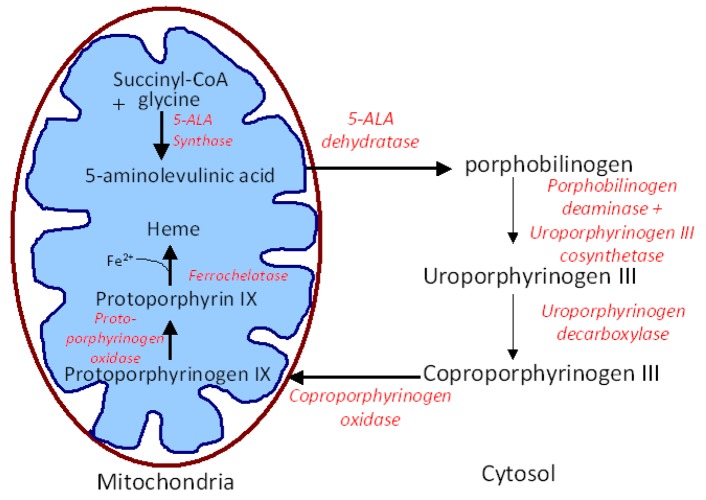
Heme biosynthesis. Schematic illustrating the interaction of the heme biosynthesis pathway with exogenous 5-aminolevulinic acid to give intracellular Protoporphyrin IX.

Fluorescence diagnosis (FD) is of increasing interest in oral cancer detection as conventional white light endoscopy that is currently used for oral cancer detection may fail to detect small and flat mucosal neoplasms, and thus are frequently overlooked during routine examination. Accurate detection and demarcation of early neoplasms followed by efficient treatment can significantly improve the survival rates of oral cancer patients. Though previous studies have evaluated the use of vital staining with Lugol's iodine and toluidine blue for improving the detection of early oral neoplasms, these methods are not yet clinically useful due to high false positive or false negative results [34,35]. FD using fluorescence endoscopy system is a technique to visualize the neoplastic lesions in a tumorous organ after topical or systemic application of a tumor-selective photosensitizer (Figure 1). Exact demarcation of tumor margins using this technique could contribute to optimum results in surgical excision and reconstruction.

Numerous photosensitizers are being investigated as fluorescent markers for *in vivo* detection and demarcation of tumors. One of the most promising photosensitizers for oral cancer diagnosis is 5-ALA. 5-ALA is a precursor in the heme biosynthetic pathway of nucleated cells. It is metabolized by certain endogenous enzymes to produced PPIX, which is an endogenous photosensitizer. PPIX is an intermediate by-product in the heme biosynthetic pathway (Figure 1) [36] and it preferentially accumulates in tumor cells due to changes in the activity of two main enzymes, porphobilinogen deaminase and ferrochelatase. Studies conducted with 5-ALA induced protoporphyrin (PPIX) fluorescence have shown a sensitivity of 95-100% for oral cancer diagnosis. Labeling of mucosal lesions of the oral cavity with PPIX fluorescence seems to be a promising diagnostic procedure for neoplastic lesions of the oral cavity [37]. 5-ALA induced PPIX fluorescence in the tissue is limited to the epithelium and both normal and dysplastic epithelium has shown PPIX fluorescence suggesting the usefulness of PPIX fluorescence in the determination of superficial tumor margins [38]. In another study, Zheng *et al.* [39] explored the application of quantifying PPIX fluorescence images to improve the diagnostic specificity and detection of early oral lesions. The results demonstrated that the combination of quantified PPIX fluorescence images with the ratio diagnostic algorithms has the potential for noninvasive diagnosis of early oral cancers *in vivo* with high diagnostic accuracy. This technique can be a useful adjunct to pathological diagnosis for directing biopsies and assessing resection margins during oral surgery.

**Figure 2.**
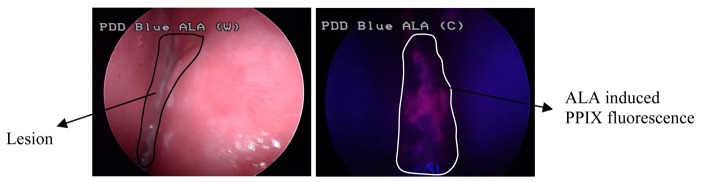
Representative images of white light (left) and ALA induced PPIX fluorescence (right) of an oral lesion acquired using a fluorescence endoscopy system.

ALA-derived PPIX fluorescence spectroscopy has been employed for the detection of epithelial hyperkeratosis (EH) or epithelial dysplasia (ED) and lesions in oral submucous fibrosis (OSF) patients that could not be detected by autofluorescence spectroscopy. The results demonstrated that ALA-induced PPIX fluorescence spectroscopy could successfully identify the premalignant lesions on oral fibrotic mucosa [40]. FD with Photofrin has been successfully used to detect hyperplastic and malignant changes in oral tissue. A quantitative analysis of the fluorescence contrast between the neoplastic and healthy tissue has established PPIX fluorescence as a sensitive, noninvasive technique for the early identification of malignant neoplasms in the oral cavity (Figure 2).

**Figure 3.**
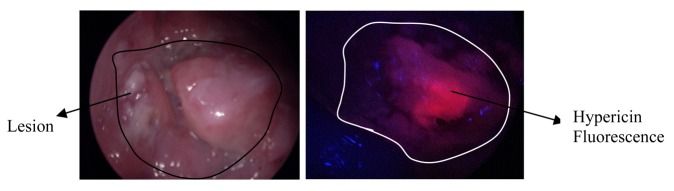
Representative images of white light (left) and hypericin image (right) of an oral lesion acquired using a fluorescence endoscopy system

Another photosensitizer, hypericin, has shown excellent fluorescence diagnostic properties in oral cavity cancers [41]. Hypericin is a plant-based photosensitizer that accumulates in abnormal cells including tumor cells [42]. Hypericin fluorescence diagnostic imaging (Figure 3) as a technique can facilitate guided biopsies in the clinic, thereby reducing the number of biopsies taken. It can also provide visualization of tumor margins during surgical procedures and assist for same-day diagnosis in the clinic. Studies have reported that hypericin fluorescence can provide improved specificity and is subject to reduced photobleaching compared to 5-ALA [43].

In both 5-ALA and hypericin fluorescence images, suspicious lesions displayed bright reddish color while normal surrounding mucosa exhibited blue color background. By quantifying the multicolor ALA fluorescence images, it was observed that the red fluorescence intensity of the malignant tissues (dysplasia, carcinoma *in situ*, squamous cell carcinoma) is much stronger than that of benign (normal, inflammation and hyperplasia) tissues, while the green fluorescence and the diffusely back-scattered blue excitation light of malignant tissue are less than benign tissue. The hypericin fluorescence images showed a progressive increase in the red to blue intensity ratios from normal to hyperplastic to SCC tissue.

However, the major disadvantage of fluorescence imaging is high background noise from auto-fluorescence from endogenous molecules such as hemoglobin and cytochromes and photodamage to biological materials. Another drawback of molecular fluorophores is their low photostability. When excited, a fluorescent molecule may actually be involved in chemical reactions, especially with oxygen free radicals thus causing photobleaching that can lead to irreversible loss of the molecule fluorescence properties. Consequently, when an assembly of fluorophores is continuously excited, their emitted light progressively fades as light induces their destruction. Even though antifading may delay this destruction, this property is a strong drawback for dynamic studies. Reducing excitation power limits the photobleaching effects and thus delays the signal decay while obviously resulting in weaker fluorescence emission. To solve these problems, upconverting fluorescent nanoparticles that emit detectable photons of higher energy in the near-infrared (NIR) or visible range upon irradiation with an NIR light in a process termed ‘upconversion’ can be employed as fluorescent labels. They overcome some of the disadvantages faced by conventional downconversion labels, thus making them an ideal fluorescent label for biological applications [44]. In addition to that, the signal weakness and photoreactivity have been partly overcome as a result of recent progress in the use of semiconductor nanocrystals, commonly called quantum dots, in biology [45]. Quantum dots emit a more intense signal and have the advantage over molecular fluorophores that they do not suffer from photodestruction. However, they introduce new problems such as blinking of the emission signal and low biocompatibility that restrict the scope of *in vivo* applications [46]. Apart from the detection of macroscopic fluorescence in tumor tissue, studies are also underway to detect microscopic fluorescence using laser confocal endomicroscopy.

## 5. Laser Confocal Endomicroscopy

**Figure 4.**
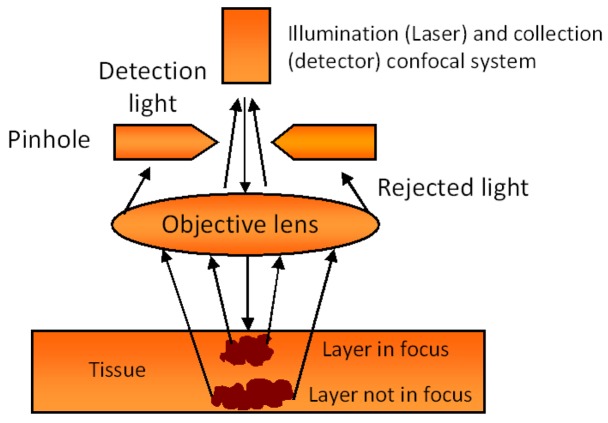
Schematic diagram of the principle of confocal endomicroscopy system.

Laser confocal endomicroscopy (LCE) is a relatively new optical technique that offers *in vivo* confocal imaging of tissue structures from surface to subsurface layers down to a few hundred micrometers (Figure 4). In our earlier study, we reported the use of a confocal endomicroscope fitted with a rigid probe for diagnostic imaging of lesions in the oral cavity [4,47]. Fluorescent dyes such as fluorescein [48] and hypericin [41], and 5-ALA, a fluorescent pre-cursor [4,47] are safe for human use and can be used for fluorescence endomicroscopic imaging in the clinic. In a pilot clinical study, ALA-induced PPIX fluorescence images from the normal human tongue were compared to images obtained from patients with squamous cell carcinoma (SCC) of the tongue. Pre-clinical studies were also carried out using rat models to compare fluorescence images from normal rat tongues to those from carcinogen-induced models of SCC. Structural images of the human and murine oral cavity obtained following ALA and fluorescein administration showed morphological differences between normal and cancer lesion. The results demonstrated the potential for laser confocal fluorescence endomicroscopy to be used as a minimally invasive optical 3D-biopsy technique for early diagnosis of oral lesions.

The laser confocal endomicroscopy system consists of a low-powered laser (an argon-ion laser that generates an excitation wavelength of 488 nm) that is focused onto a single point in a defined microscopic field of view, and the same lens is used as both the condenser and objective folding optical path. The point of illumination thus coincides with the point of detection within the specimen. Light emanating from that point is focused through a pinhole to a detector, and light emanating from outside the illuminated spot is rejected. The illumination and detection systems are in the same focal plane and are termed “confocal”. Because confocal images depend on fluorescence, a fluorescent dye (contrast agent) is required to make objects visible. All detected signals from the illuminated spot are captured and measured. The gray-scale image created is an optical section representing one focal plane within the examined specimen. The image of a scanned region can be constructed and digitized by measuring the light returning to the detector from successive points. Series of confocal images within successive planes can be used to observe fine cellular or subcellular structures, and three-dimensional structures in the specimen can be imaged [49].

**Figure 5.**
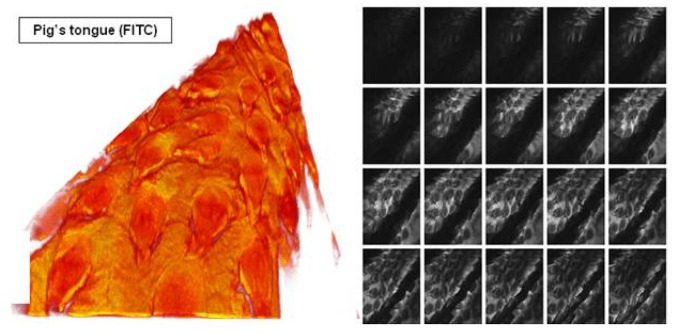
Reconstruction of optical slices stack to achieve 3D visualization of pig tongue using FITC as fluorescence contrast agent has been demonstrated.

Recently, Haxel *et al.* extended the use of confocal endomicroscopic imaging to five regions of the human oropharynx, namely the buccal area, the tongue, the base of the tongue, the tonsils, and the floor of the mouth [50]. *In vivo* imaging was carried out using intravenously injected fluorescein while the dye acriflavine was used for *ex vivo* imaging of SCC tissue. Their results showed that confocal endomicroscopy is suitable for epithelial evaluation of the anterior parts of the oral cavity, including the buccal area, tongue and floor of the mouth. The technique however was not suitable for application on the tonsils or the base of the tongue as the procedure triggered pharyngeal reflexes in the subjects examined. Our recent results have demonstrated the potential for laser confocal fluorescence endomicroscopy to be used as a minimally invasive optical 3D-biopsy technique for early diagnosis of oral lesions. In Figure 5, reconstruction of optical slices stack to achieve 3D visualization of pig tongue using FITC as fluorescence contrast agent has been demonstrated.

Though fluorescence imaging has long been the technique of choice to map the biochemical distributions in a living system, current cell biophysics research has generated great interest owing to its sensitivity and ease of use. This is because fluorescence signals emanated from both the intrinsic bio-molecules as well as the exogenously introduced fluorophores, may lack specificity due to overlapping signals between different fluorescent species. In addition, photobleaching that may occur as a result of the fluorophores undergoing exhaustive absorption/emission cycles is also a limiting factor in using the fluorescence technique.

The disadvantages of confocal endomicroscopy include the fact that it is an examiner-dependent technology and thus hands-on training is of crucial importance before reliable histological diagnosis can be made. *In vivo* imaging using LCE involves consumption of considerably long duration of time. Also the system requires manual control fully operated by the user. There are also imaging flaws that affect the image quality. Apart from that, the lack of a realtime volume visualization system limits the flexibility for changes on-the-spot during imaging [51]. Consequently, it is meaningful to explore various ideas and methods proposed to view 3D datasets as 2D images. It is also clinically useful if the captured slices can be perceived from arbitrary viewing angles or highlight desired features. These functions introduce user interaction and further promote the effectiveness of visualization [52].

Numerous fluorescence dyes have been tested with LCE imaging. Of the potentially suitable contrast agents in humans, the most commonly used are intravenous fluorescein sodium (10%) and topically applied acriflavine (0.2%) [49]. Common side effects include allergic reactions with a drop in blood pressure or cardiac rhythm abnormalities (0.6%), nausea (3.5%) or local complications such as extravasation and/or thrombophlebitis (0.16%) [53]. Use of intravenous fluorescein for gastrointestinal LCE has been shown to be safe with few acute complications [54]. Fluorescein-aided LCE of the lung appeared to be safe and well tolerated. While the lack of staining of cells in the central airways was a major limitation, it permitted analysis of the lung interstitium and alveolar space and thus emerges as a new approach for the *in vivo* analysis of interstitial lung diseases [55]. Fluorescein sodium is a nonspecific vascular contrast agent that binds to serum albumin, enhancing visualization of vascular structures. Unbound fluorescein escapes the vascular space to enhance visualization of the extracellular matrix; however, it does not penetrate cells to provide imaging of the cell nucleus, which is a major limitation of current LCE imaging. Imaging the nuclei is essential to differentiate between high-grade dysplasia and low-grade dysplasia because these are important criteria in the current Vienna classification of gastrointestinal (GI) epithelial neoplasia [56]. Therefore, the development of fluorescent markers that either selectively label cells that are dysplastic or cells, allowing visualization of the cell nuclei, has the potential to significantly improve the diagnostic capability of LCE.

Topically administered acriflavin stains cell nuclei of the surface epithelium but does not penetrate to deeper layers of the GI mucosa [57]. Acriflavin is a mutagenic dye and a potential human carcinogen, which will likely limit its clinical utility [58]. Novel fluorescent contrast agents are being developed for LCE imaging to target disease-specific biomarkers. These include labeled peptides that can be easily delivered to the target site due to its low molecular weight, but they have variable affinity [59]. Another study reported the topical use of heptapeptide conjugated with fluorescein for specific *in vivo* imaging of human colorectal neoplasia [60]. Fluorescently labeled antibodies can be highly selective, binding to their defined target, but may induce immune reactions [59]. Using LCE human xenograft tumors in mice and human tissue specimens could be effectively classified based on their EGFR expression using fluorescently labeled antibodies against EGFR [61]. Moreover, anti-VEGF antibodies have been used as fluorescent contrast agents for *in vivo* molecular imaging in animal models [62]. However, extensive pharmacokinetic and safety studies are needed to define clinical applications for these molecular probes. Therefore, imaging techniques such as Raman spectroscopy is being modified and explored to provide molecular information for oral cancer detection.

## 6. Surface Enhanced Raman Spectroscopy

Surface Enhanced Raman Spectroscopy (SERS) is a Raman Spectroscopic (RS) technique that provides dramatically amplified Raman signals from Raman-active analyte molecules that have been adsorbed onto certain roughened noble metal surfaces [63]. RS is ineffective for surface studies because the photons of the incident laser light simply propagate through the bulk and the signal from the bulk overwhelms any Raman signal from the analytes at the surface. Fortunately, such a disadvantage can be circumvented by the use of SERS. In SERS when the nano-structured metallic substrates are made to resonate with external optical fields, oscillating surface plasmons are produced [64,65]. This brings about extremely intense local electric fields at certain sites, known as the SERS hot-spots, on the metal surface, causing the adsorbed molecules to experience tremendous excitation fields, and hence the subsequent emission of strong Raman signals (12, 24). It has been shown that such a resonant response can greatly enhance the efficiency of Raman scattering, typically by up to 10^6^ to 10^7^ fold [66]. Further improvement in the enhancement factor by a factor of 10^4^ to 10^5^ is also possible if the molecules are sandwiched in between particles within an aggregate, giving the possibility of single-molecule spectroscopy [67]. With such a high sensitivity and chemical specificity, SERS are beginning to gain popularity as a powerful optical tool for the quantitative multi-component analysis of cells or tissues, and the present review intends to summarize the art of SERS in the domain of analytical spectroscopy and spectral imaging.

Using Fourier-transform (FT)-Raman spectroscopy, Oliveira *et al.* [68] demonstrated the changes in the vibrational bands of normal, dysplastic (DE) and squamous cell carcinoma (SCC) tissues that seem to arise from the compositional, conformational and structural changes of proteins. Another study has shown that principal components analysis (PCA) of the Raman spectral data can discriminate between normal, inflammatory, premalignant, and malignant conditions in oral tissue [69]. Huang *et al.* [70] demonstrated the applications of anti-EGFR conjugated Au nano-rods for *in vitro* SERS spectroscopic analysis of cell surface on a malignant epithelial cell line, HSC 3 (human oral squamous cell carcinoma) cultured on a cover slip. Although not reported by Huang *et al.* in his study, it would be worthwhile to compare the SERS spectra from non-malignant and malignant cells since the chemical composition of cell membrane is known to undergo alterations in cancerous cells. Qian *et al.* [71] on the other hand, demonstrated the use of SERS tags for *in vivo* targeting of human head-and-neck tumor (Tu686) in nude mice at the near infrared region (785 nm). In order to achieve this, pegylated Au nanoparticles bearing malachite green SERS reporter and covalently attached ScFv antibody was injected systemically through tail vein into the tumor bearing nude mice. Spectra derived from the tumor site indicated that the antibody conjugated Au nanoparticles were able to target the tumor *in vivo*.

**Figure 6.**
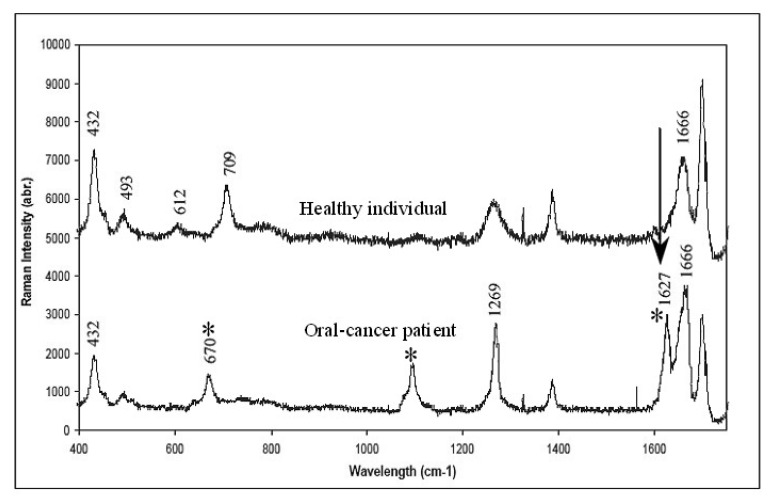
SERS spectra of raw human saliva samples. * highlights the differences in the spectra of oral cancer patients when compared with healthy individual (From Reference [5]).

In our effort to develop non-invasive imaging for oral cancer diagnostics we investigated the possibility of SERS to detect specific molecular markers in the saliva of oral cancer patients. Three Raman peaks that were noticed at 670, 1079, and 1627 cm^−1^ are known to be the characteristics of saliva specimens collected from cancer patients (Figure 6). Of these bands, special note must be given to that situated at 1627 cm^−1^, which were observed for 3 of the 5 saliva samples collected from the patients. This particular band was also observed in a previous Raman study of cancerous oral tissues [72]. As to whether there is any correlation in the Raman signals at 1627 cm^−1^ between the saliva spectra and those derived from the tissues is not known at the moment. A comparison of SERS saliva spectra and the Raman spectra derived from matched oral tissues that is currently being performed in our lab would certainly provide an answer. We are also unable to evaluate the applicability of using the saliva spectrum for cancer staging at this stage, owing to insufficient samples. From further patient samples, we observed a sensitivity of the current technique in the range of around 70%, *i.e.*, 70% of the “abnormal” saliva shows abnormal peaks. However, none of the normal samples show these indicative peaks. Currently work is underway to develop a better SERS substrate consisting of periodic metallic nanostructures as opposed to the randomly distributed nanoparticles film that was employed in this study. This would improve signal reproducibility, thereby rendering quantitative analysis of the absolute signal intensity possible. However, the results of this study show the potential of these simple gold nanoparticles film to be used in routine clinical diagnosis of oral cancer.

In our earlier study, we demonstrated the use of gold nanoparticles in surface enhanced Raman scattering to enhance the Raman spectroscopy signal for the analysis of cancer-related chemical changes in saliva [5,73]. We developed a simple and cost-effective method for preparing highly sensitive saliva assay based on SERS-active gold nanoparticle films and used to analyze biofluids spectroscopically, the SERS from the saliva biofilm interface seeks spectral features indicative of oral cancer. The use of saliva as a diagnostic fluid would offer huge advantages over previous sera-based counterparts in that saliva is easily accessible, painlessly acquired and presents lower risk of infection compared to serum.

When evaluated in clinical studies, Raman spectroscopy still suffers the significant disadvantages of being random and nonimaging [74]. Nevertheless, Raman and FLIM signals are weak and require expensive equipment, extensive process and complex algorithms to differentiate tissue types. Another limitation of non-imaging spectroscopic techniques is the lack of spatial information, which is very essential for examining complex biological tissues. These issues present challenges for their future clinical use. Merging of imaging and spectroscopy can overcome this limitation [75].

Next we will focus on optical coherence tomography, which is another imaging technique that is designed to image cell and stromal morphology for non-invasive clinical diagnosis in real time without the need for needle biopsy.

## 7. Optical Coherence Tomography

Optical coherence tomography is a non-invasive optical imaging technique that produces cross-sectional images of tissue with a high spatial resolution of 10 to 20 μm [76]. It uses broadband-limited coherence range laser light in near-infrared (NIR) region (770 to 1300 nm) to scan the topology of the tissue subsurface [77]. This is the optical window of biological tissue where predominant light absorption and scattering can be minimized to achieve adequate light penetration for optical imaging. The inevitable light attenuation in biological tissues often limits penetration depths range from 1 to 2 mm [78]. However, epithelial cancers are likely to occur within 600 μm of the tissue surface, which is an ideal imaging depth for OCT imaging systems. With this range of tissue penetration depth, OCT technology is well suited for imaging oral mucosa [72,79] as the normal human oral mucosa is very thin, ranging from 0.2 to 1 mm [80].

**Figure 7.**
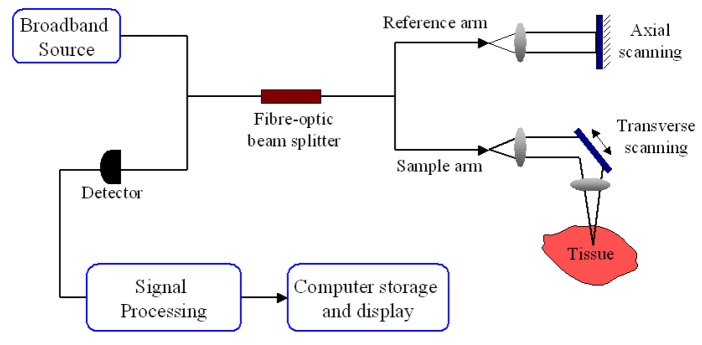
Schematic diagram for Time-Domain-optical coherence tomography.

A typical OCT setup (Figure 7), *i.e.*, time-domain OCT (TD-OCT), for tissue probing is based on a Michelson interferometer configuration. Illumination light beam is split equally to the reference arm and sample arm. The reference arm consists of a movable mirror that gives the depth information of the image while the sample arm directs the beam to the sample to acquire the lateral image information. The backscattered signal from tissue is later interfered with the reflected reference beam. The interfered signals are measured by a detector to construct the depth profile of tissue.

The use of OCT for studying abnormal structure in the soft and hard tissue of the oral cavity was first introduced in 1998 [81,82]. Since then extensive *in vivo* and *ex vivo* OCT imaging has been primarily conducted on a hamster cheek pouch model to study the epithelial and sub-epithelial changes in oral carcinogenesis [72,83]. In addition to the mapping of structural changes, OCT in Doppler mode, *i.e.*, Optical Doppler Tomography, is also capable of quantifying the velocity of blood flow, particularly useful in detecting vascular changes associated with carcinogenesis [84]. With the extended numerical aperture and penetration depth of this OCT configuration, it is feasible to determine the scattering coefficient of the nucleus, which was found to increase as the normal cells progress to invasive cancer stage [83]. Later, *in vivo* 3-D OCT was available to visualize the detailed histological features of the oral lesions with an imaging depth down to 2–3 mm. The comprehensive diagnostic images can be used to define surgical margin and to provide a direct assessment of the therapeutic effectiveness. Furthermore, OCT system can be readily combined with nonlinear optical modalities, such as two-photon excited fluorescence (TPF) and second-harmonic generation (SHG). The combined techniques were found to yield increased sensitivity and specificity in the diagnosis of oral dysplasia and malignancy [84]. Subsequently, the investigation of OCT extended to human clinical studies [85]. Fiber-based OCT probe was customized and combined with an operative endoscopy to facilitate the imaging of oral cavity and oropharynx of 41 patients [85]. Recently, a human clinical study of 50 patients was carried out to substantiate the ability of OCT for diagnosing oral dysplasia and malignancy [79]. They demonstrated the ability of OCT to evaluate the macroscopic morphology of keratin, epithelium, sub-epithelium and basement membrane at near histopathological-level resolution. The results showed that there is a strong agreement between OCT-based diagnosis and histopathological diagnosis with sensitivity and specificity approximately around 0.93–0.97.

Several types of particulate contrast agents such as air-filled microbubbles, engineered microspheres and gold nanostructures have been developed to improve the OCT image by enhancing the intensity of backscattered light from the tissue [86,87]. Amongst these, gold nanostructures of different types e.g. gold nanorods [88], gold nanocages [89] and gold nanoshells [8] have been the most widely studied. The choice of gold nanostructures as OCT contrast agents is motivated by several factors: (i) the known biocompatibility of gold; (ii) the small size of the nanoparticles that increases their portability in tissue; (iii) the easy tunability of their optical properties; and (iv) their high scattering efficiencies (>1) arising from the surface plasmon resonance of gold at the nanoscale compared to dielectrics such as polystyrene which must be micron-sized to achieve efficiencies near unity.

OCT can provide resolutions approaching that of conventional histopathology. Cellular-level image resolutions as fine as 1 μm have been demonstrated in biological specimens, allowing visualization of the mitotic cycle and tracking cell migration. Ultrahigh resolutions of 3 μm have been achieved in ophthalmic imaging, enabling visualization of internal retinal architectural morphology and promising to improve the accuracy and reproducibility of retinal morphometry [90]. OCT provided test outcomes for differentiation between benign laryngeal lesions and dysplasia/CIS with sensitivity of 88%, specificity of 89% and predictive accuracy of 88%. OCT allows for a fairly accurate assessment of benign and dysplastic laryngeal epithelial lesion and greatly facilitates the taking of precise biopsies [91]. However, there are several issues related to the use of OCT for clinical studies. These include the fact that: (i) a histopathologist is required to interpret and assess the live image. interpretation and assessment of the acquired live histology images; (ii) as the images do not provide quantitative information, visual assessment would be needed, which is subjective; (iii) due to the small size of the OCT probe, only a very small area can be examined at a time. Therefore, the detection of functional and structural changes at a precancerous stage still remains a challenge for OCT [75].

## 8. Confocal Reflectance Microscopy

*In vivo* confocal reflectance microscopy (CRM) is an optical tool to noninvasively image tissue with near histological resolution [92]. Image contrast is determined by natural differences in refractive indices of organelles and other subcellular structures within the tissues [93]. CRM differs from a conventional microscope as it uses a laser to illuminate a small spot within tissue. Light that is backscattered from the tissue is captured through an aperture, which matches the size of the illuminated spot placed in front of the detector. The aperture spatially filters light returning from out-of-focus planes within the tissue, such that only the plane in focus is imaged. CRM can therefore image viable tissue with high-resolution and contrast without performing a biopsy and processing the tissue, as in standard histology.

Basically the CRM set-up consists of a light source, a condenser, an objective lens and a detector. A tightly focused beam of light is focused into a small spot at a chosen depth within the volume of a sample. Only the light from the chosen spot at the chosen depth is in focus at the pinhole and is able to pass through it unimpeded. By moving the beam in one direction (x-axis), a line of reflected signals is generated. By moving the beam in the other direction (y-axis), a complete area can be scanned leading to an en face image of the tissue. That is comparable to an “optical slice” of the tissue. By moving that plane into or out of the tissue (z-axis), a stack of images can be generated. This property enables a CRM to look at a slice of a thick semi-transparent sample, whereas, conventional microscopes visualize all the planes contemporarily [94,95].

A study by White *et al.* has demonstrated the imaging of lip and tongue mucosa using CRM in healthy patients [96]. CRM provided details of cells and organelles including nuclei, circulating blood cells and extracellular matrix without the artifacts of histological processing. Characteristic histologic features of different tissues in the head and neck and cell types were readily discernible by CRM and correlated well with permanent sections. However, compared to paraffin-embedded histological sections, less microscopic details were visible in the CRM images [93]. Another study reported the use of fiber optic confocal reflectance microcope to diffentiate normal and neoplastic tissues in oral muscosa [97]. Distinct features such as nuclear irregularity and spacing, which can be used to qualitatively differentiate between normal and abnormal tissue were identified. In the same way, confocal laser scanning microscope is also suitable for noninvasive *in vivo* examination of the tongue. On the tongue surface both filiform and fungiform papillae and their taste pores could be easily identified. The epithelium of the tongue with its subcellular structures could be observed up to a depth of 50 μm, cellular structures up to 150 μm and subepithelial vessels up to 300 μm. Additionally the papillary crests and blood flow were also visible [98]. On the other hand, a rigid confocal endoscope that was developed to assess the oral squamous epithelium of mice could image the underside of the tongue at a penetration depth between 104 and 240 μm. For differentiation between low-grade and high-grade squamous intra-epithelial lesions, a sensitivity and specificity of 73% and 88% was reached [99].

Apart from oral mucosa, CRM has been used to image human dermis. Imaging of human epidermis and superficial dermis using reflectance confocal imaging can exploit inherent differences in the reflectivity of microstructures within and between cells to produce contrast, at specific illumination wavelengths [100,101]. Thus far, confocal reflectance imaging has demonstrated significant promise for clinical applications such as guiding skin biopsy, determining the margins of skin cancers to guide surgical excision, noninvasive monitoring of the efficacy of laser and topical drug treatments, and sensitivity and specificity studies of screening and diagnosis of skin cancer [102].

CRM is an optical method of noninvasively imaging tissue without fixation, sectioning, and staining as in standard histopathology thus has the potential for further development and use in clinical practice for pre-surgical determination of cancer margins and intra surgical guidance on the patients in real time. This it can act as a diagnostic tool for the early detection of oral cancer and precancer.

## 9. Conclusion

Early detection of oral premalignant lesions and early neoplastic changes may be the most effective means to improve survival and quality of life for oral cancer patients. The main contribution of autofluoresence, confocal reflectance imaging and fluorescence imaging is to highlight oral lesions and to assist the physicians to better locate the surgical margins. Further development of SERS with improved metallic nanostructure substrates can be useful in the detection of specific molecular markers in the saliva of oral cancer patients. Furthermore, the strong agreement between OCT-based diagnosis and histopathological diagnosis reported in this article supports the concept that OCT can be a very useful tool for the early detection and diagnosis of oral lesions. As the technology and techniques evolve, imaging modality may progressively reduce the need for biopsy, define surgical margins, and provide a direct evaluation of the effectiveness of oral cancer. A diagnostic technique capable of performing optical biopsy for noninvasive pathological diagnosis of cancer *in situ* and in real time should prove to be a powerful diagnostic modality in clinical medicine that will likely be a significant clinical advance with considerable impact on patient management.
